# Amaurosis Fugax in Posterior Reversible Encephalopathy Syndrome: A Vexed Hurdle in a Postpartum Primigravida Patient

**DOI:** 10.7759/cureus.43703

**Published:** 2023-08-18

**Authors:** Abhinav Ahuja, Keyur Saboo, Sunil Kumar, Sourya Acharya, Sachin Agrawal

**Affiliations:** 1 Department of Medicine, Jawaharlal Nehru Medical College, Datta Meghe Institute of Higher Education and Research, Wardha, IND

**Keywords:** posterior reversible encephalopathy syndrome, posterior leukoencephalopathy syndrome, amaurosis fugax, eclampsia, pregnancy

## Abstract

In this case report, we highlight a case of a 24-year-old primigravida who suffered a sudden and painless loss of vision and headache in the immediate postpartum period. Vision loss was transient and remarkable. Her brain magnetic resonance imaging revealed vasogenic edema in parieto-occipital white matter, suggestive of posterior reversible encephalopathy syndrome. Posterior reversible encephalopathy syndrome is a clinical-radiological entity, having hemodynamic catastrophe also known as reversible posterior cerebral edema syndrome. It tends to occur during pregnancy complicated by eclampsia. Hypertension and a hypercoagulable tendency tend to engulf the entire homeostasis into its deadly clutches sending the autoregulation into a frizzy. It presents with a gamut of red flags like headache, seizures, encephalopathy, amaurosis fugax, cortical visual disturbances, and even blindness. Clinical improvement was seen with supportive treatment in this patient. Thus, timely diagnosis and intervention help reverse the dire consequences.

## Introduction

The occipital and posterior parietal lobes of the brain are both affected by the neurologic condition known as posterior reversible encephalopathy syndrome (PRES). The majority of patients (over 70%) have hypertension; however, some also have normal or only slightly increased blood pressure [1]. Headaches, convulsions, visual abnormalities, and altered sensorium are frequent presentations [2]. Blurring of vision, homonymous hemianopsia, and even cortical blindness are all on the visual symptom spectrum [1]. Numerous additional symptoms, including nausea, vomiting, and abnormalities in the brain stem, have also been documented [1,2]. Acute kidney injury, hypertensive encephalopathy, cytotoxic medications, and autoimmune diseases have all been linked closely to PRES. Neuroimaging reveals alterations in the parieto-occipital white matter [3,4]. The loss of vision in one or both eyes that typically lasts four to 60 minutes is referred to as "amaurosis fugax" [3,4]. This symptom is typically caused by a thromboembolic condition, in which carotid artery or aortic arch atherosclerotic plaque fragments temporarily restrict blood flow in the branch or central retinal arteriolar network [5]. The hemodynamic condition is unstable throughout pregnancy, which increases the risk of preeclampsia, eclampsia, and severe pregnancy-induced hypertension (PIH). The occurrence of PRES in these women who arrive with a rapid loss of vision and neurological symptoms must be questioned as early identification and treatment can be extremely beneficial and prevent the patient from serious morbidities. Amaurosis fugax is the term used to describe monocular or binocular vision loss that typically lasts four to 60 minutes. The carotid artery or the aortic arch-originating atherosclerotic material that momentarily blocks blood flow in the branch or central retinal arteriolar network is the most common source of this symptom's genesis.

## Case presentation

**Figure 1:**
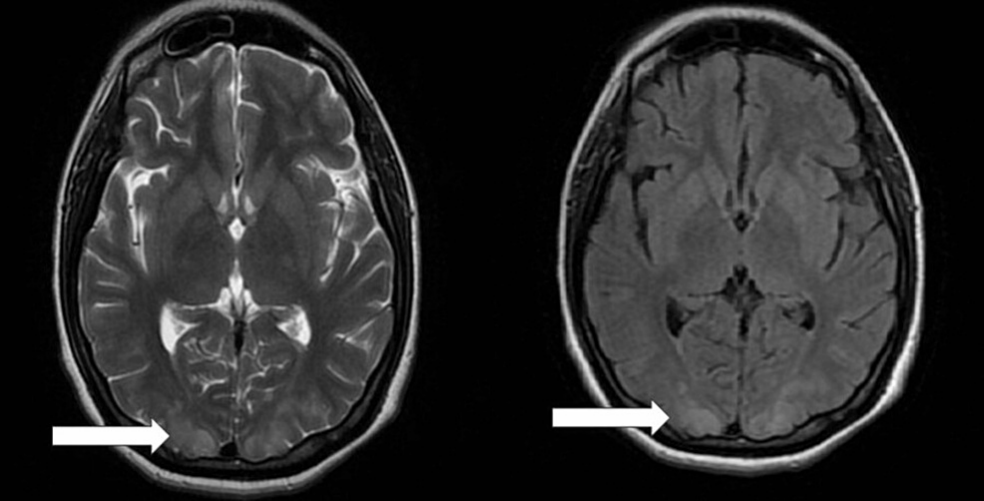
Magnetic resonance imaging of the brain showing subcortical areas of altered intensity indicated by the white arrow.

A 24-year-old primigravida presented on the third postpartum day with complaints of a sudden onset of painless loss of vision and severe headache since one day. She had delivered outside in a local hospital. There was no past history of hypertension, visual problems, bronchial asthma, cardiac disease, or seizures. Vision in both eyes was counting fingers at one meter. On fundoscopy, the optic disc was normal, and there was no evidence of papilloedema. Blood pressure was 170/100, pulse was 80/minute, and respiratory rate was 22/minute. The patient was afebrile. The chest was clear on auscultation. The patient was conscious and well-oriented. Other system examination results were likewise normal, and bilateral plantar reflexes were flexor. The electrocardiogram indicated a typical sinus rhythm. The MRI of the brain clearly indicates a significant alteration in intensity within the subcortical regions of both posterior occipital lobes (Figure 1). All other laboratory parameters are within normal limits (Table 1). The patient was given intravenous mannitol 100 ml thrice a day for three days, nitroglycerine (NTG) injection infusion at a rate of 0.3 ml/hr for the management of cerebral edema, and blood pressure was given for three days. After four days of hospitalization, the patient showed remarkable improvement in her symptoms and signs. Vision in both eyes improved to 6/9, and she was also relieved of headaches. The drastic improvement in vision was a remarkable finding. The rest of her stay in the hospital was uneventful. The patient was discharged after eight days of presentation to the hospital, and on follow-up after one month, the patient had no clinical symptoms.

**Table 1:** Laboratory parameter of the patient with reference range.

Investigations	Patient	Reference Values
Hemoglobin	11.7 g/dl	13-17 g/dl
Total leukocyte count	8,200 /dl	4,000-11,000/dl
Platelet count	453,000/dl	150,000-400,000/dl
Serum creatinine	0.5mg/dl	0.5-1.2 mg/dl
Albumin	4.8 g/dl	3.5-5.0 g/dl
Aspartate aminotransferase	38 U/L	<50 U/L
Alanine aminotransferase	50 U/L	17-59 U/L
Total bilirubin	0.7 mg/dl	0.2-1.3 mg/dl
Homocysteine	11.83 mmol	6.6-14.8mmol
Erythrocyte sedimentation rate	45 mm/hr	0-20 mm/hr
International normalized ratio	1.10	0.8-1.1
Prothrombin time	12.1	11.9
Activated partial thromboplastin time (APTT)	31.8	29.5

## Discussion

We find ourselves in a difficult situation when a postpartum primigravida presents with an abrupt loss of vision. There are a number of possibilities, including eclampsia, posterior reversible encephalopathy syndrome (PRES), and cerebrovascular events complicating pregnancy [6]. Visual consequences of eclampsia-complicated pregnancy include cortical blindness, exudative retinal detachment, and hypertensive retinopathy [7]. Normal fundoscopy and intact pupillary reflexes are indicators of cortical blindness. As PRES can be reversed, eyesight can be recovered in four to eight days [7]. The patient’s visual symptoms can be very concerning. According to Lifson et al.'s study, patients with PRES-related visual problems frequently make a full recovery after receiving treatment. It is anticipated that those whose deficiencies persist will see a significant improvement in their eyesight compared to how they initially appeared [8].

The pathogenesis of PRES is poorly understood, but the most accepted theory is vasogenic edema [1]. Balanced homeostasis is pivotal in regulating the organ systems of the body. However, during pregnancy, particularly eclampsia, this homeostasis is thwarted and thus leads to a cascade of pathological events causing disruption of autoregulation in the brain. Vasogenic edema in the brain is brought on by endothelial damage and disturbance of the blood-brain barrier. For the patient, this might be disastrous. Neuroimaging white matter hypodensity is visible on computed tomography in the parieto-occipital areas, although MRI of the brain is the gold standard for the diagnosis of PRES. This can be explained by the fact that the anterior circulation of the brain has higher autoregulation than the posterior circulation due to better sympathetic innervation [9].

Even in asymptomatic people, finding Hollenhorst plaques during a routine eye exam can sometimes be a warning of serious thromboembolic illness; this is especially true if a carotid bruit can be heard during auscultation. The evaluating clinician must auscultate the carotid artery in addition to taking into account advanced imaging modalities of the carotid arteries and occasionally the aorta and its branches because this patient had culprit atherosclerotic plaques. Aortic or carotid atherosclerosis in a patient with amaurosis fugax requires prompt administration of antiplatelet medicine and a statin. This complies with the suggested procedures. Amaurosis fugax is most usually associated with significant internal carotid artery atherosclerosis, yet there should still be some concern regarding atheroembolism from the aorta or its branches, including the subclavian or brachiocephalic arteries [10,11].

Reversibility of symptoms on the initiation of treatment of elevated blood pressure and withdrawal of offending agents aids to confirm the diagnosis of PRES. Our patient presented with a marked decrease in vision and a severe headache. MRI showed bilateral occipital lobe edema. However, the resolution of edema and reversibility of symptoms on treatment helped us clinch the diagnosis of PRES, and it gives an important insight into the diagnosis and how to differentiate it from other medical conditions. Amaurosis fugax in pregnant hypertensive women must compel us to think about PRES.

Thus, timely diagnosis with a high order of suspicion and intervention helps to reverse the dire consequences. However, a few undesirable tell-tale signs do remain.

## Conclusions

Pregnant women who report sudden vision impairment, high blood pressure, and hemodynamic problems can be diagnosed with posterior reversible encephalopathy syndrome. Medical professionals should take note of this sobering example of amaurosis fugax in a patient with posterior reversible encephalopathy syndrome and be alert for odd neurological illness symptoms. Early detection, careful examination, and accurate diagnosis are necessary for the prompt and proper delivery of therapy, which ultimately improves patient outcomes and quality of life, neurological deficits may become permanent or fatal if treatment is insufficient or delayed. Our understanding of PRES will also improve with continued reporting and sharing of such cases, which will aid in the development of evidence-based recommendations for its diagnosis and treatment. It is imperative that healthcare workers utilize the knowledge acquired from this experience in comparable circumstances that may arise in the future.
